# Hypotension prediction index for prevention of intraoperative hypotension in patients undergoing general anesthesia: a randomized controlled trial

**DOI:** 10.1186/s13741-024-00414-7

**Published:** 2024-06-15

**Authors:** Chih-Jun Lai, Ya-Jung Cheng, Yin-Yi Han, Po-Ni Hsiao, Pei-Lin Lin, Ching-Tang Chiu, Jang-Ming Lee, Yu-Wen Tien, Kuo-Liong Chien

**Affiliations:** 1 Institute of Epidemiology and Preventive Medicine, National Taiwan University, No. 17, Xu-Zhou Rd, Taipei, 10055 Taiwan; 2 Department of Anesthesiology, National Taiwan University Hospital, Taipei, Taiwan; 3 Department of Traumatology, National Taiwan University Hospital, Taipei, Taiwan; 4 Division of Thoracic Surgery, Department of Surgery, National Taiwan University Hospital, Taipei, Taiwan; 5 Division of General Surgery, Department of Surgery, National Taiwan University Hospital, Taipei, Taiwan; 6 Department of Internal Medicine, National Taiwan University Hospital, Taipei, Taiwan; 7 Population Health Research Center, National Taiwan University, Taipei, Taiwan

**Keywords:** General anaesthesia, Hypotension prediction index, Intraoperative hypotension, Postoperative complications, Time-weighted average mean arterial pressure

## Abstract

**Background:**

Intraoperative hypotension is a common side effect of general anesthesia. Here we examined whether the Hypotension Prediction Index (HPI), a novel warning system, reduces the severity and duration of intraoperative hypotension during general anesthesia.

**Methods:**

This randomized controlled trial was conducted in a tertiary referral hospital. We enrolled patients undergoing general anesthesia with invasive arterial monitoring. Patients were randomized 1:1 either to receive hemodynamic management with HPI guidance (intervention) or standard of care (control) treatment. Intraoperative hypotension treatment was initiated at HPI > 85 (intervention) or mean arterial pressure (MAP) < 65 mmHg (control). The primary outcome was hypotension severity, defined as a time-weighted average (TWA) MAP < 65 mmHg. Secondary outcomes were TWA MAP < 60 and < 55 mmHg.

**Results:**

Of the 60 patients who completed the study, 30 were in the intervention group and 30 in the control group. The patients’ median age was 62 years, and 48 of them were male. The median duration of surgery was 490 min. The median MAP before surgery presented no significant difference between the two groups. The intervention group showed significantly lower median TWA MAP < 65 mmHg than the control group (0.02 [0.003, 0.08] vs. 0.37 [0.20, 0.58], *P* < 0.001). Findings were similar for TWA MAP < 60 mmHg and < 55 mmHg. The median MAP during surgery was significantly higher in the intervention group than that in the control group (87.54 mmHg vs. 77.92 mmHg, *P* < 0.001).

**Conclusions:**

HPI guidance appears to be effective in preventing intraoperative hypotension during general anesthesia. Further investigation is needed to assess the impact of HPI on patient outcomes.

**Trial registration:**

ClinicalTrials.gov (NCT04966364); 202105065RINA; Date of registration: July 19, 2021; The recruitment date of the first patient: July 22, 2021.

**Supplementary Information:**

The online version contains supplementary material available at 10.1186/s13741-024-00414-7.

## Introduction

Intraoperative hypotension is a common side effect of general anesthesia, and it can lead to inadequate organ perfusion (Ackland & Abbott 2022). An increased risk has been noted when the mean arterial pressure (MAP)s falling below 60 to 65 mm Hg for prolonged periods or below 50 to 55 mm Hg for any amount of time (Wesselink et al.^2018^). In patients undergoing general anesthesia, hypotension can trigger postoperative stroke, acute kidney injury and myocardial injury by compromising blood flow to ischemic areas (Bijker et al.^2012^; Salmasi et al.^2017^; Tang et al.^2019^; Walsh et al.^2013^). Further, intraoperative hypotension is linked to extended hospital stays, higher postoperative surgery-related morbidity, and potentially even mortality (Bijker et al.^2009^; Monk et al.^2015^; Südfeld et al.^2017^; Temesgen et al.^2021^). Predicting intraoperative hypotension remains challenging. If clinicians were able to shift from reactive management to a more proactive strategy and treat intraoperative hypotension before it occurs, harm could be significantly reduced.

The Hypotension Prediction Index (HPI) is a novel parameter (Edwards Lifesciences, Irvine, CA, USA) ranging from 0 to 100, with a higher HPI indicating a greater probability of impending hypotension (Davies et al.^2020^; Hatib et al.^2018^). The algorithm used to calculate the HPI can predict a hypotensive event, which is defined as mean arterial pressure (MAP) < 65 mmHg for ≥ 1 min, up to 15 min before the event (Davies et al.^2020^; Hatib et al.^2018^; Maheshwari et al.^2020^). In addition to providing an early warning, the HPI can offer other advanced hemodynamic information, such as cardiac output, dynamic arterial elastance, systolic slope, and stroke volume, to help determine the underlying cause of the impending hypotension (Maheshwari et al.^2020^). However, previous studies have not reached a consensus on whether HPI guidance can reduce the duration and severity of intraoperative hypotension and prevent postoperative complications (Maheshwari et al.^2020^; Murabito et al.^2022^; Šribar et al.^2023^; Tsoumpa et al.^2021^; Wijnberge et al.^2020^).

The aim of this study was to evaluate whether HPI guidance can effectively reduce the severity and duration of intraoperative hypotension during general anesthesia. To test our hypothesis, we conducted a randomized controlled trial comparing the severity of intraoperative hypotension between patients receiving HPI guidance and those receiving standard care (without HPI guidance). In addition, we also tested the threshold of 60 and 55 mmHg for intraoperative hypotension.

## Methods

This single-center randomized, controlled trial was conducted at a tertiary referral hospital from July 22, 2021 to January 26, 2022.

### Inclusion and exclusion criteria

We recruited adult patients between 20 and 80 years of age, who were planning to receive elective non-cardiac surgery with general anesthesia, which required continuous invasive blood pressure monitoring via an arterial catheter. The target MAP for all patients was at least 65 mmHg. We excluded patients 1) who had undergone emergency procedures; 2) with known clinically important cardiac disease, such as moderate to severe valvular disease; 3) with a need for a tidal volume < 8 mL/kg of ideal body weight during surgery; and 4) with current persistent atrial fibrillation (Maheshwari et al.^2020^). After initially evaluating eligible patients during their preoperative visit and obtaining their written informed consent, we enrolled patients who were undergoing general anesthesia that was expected to last for > 4 h.

### Randomization, allocation and blinding

**Fig. 1:**
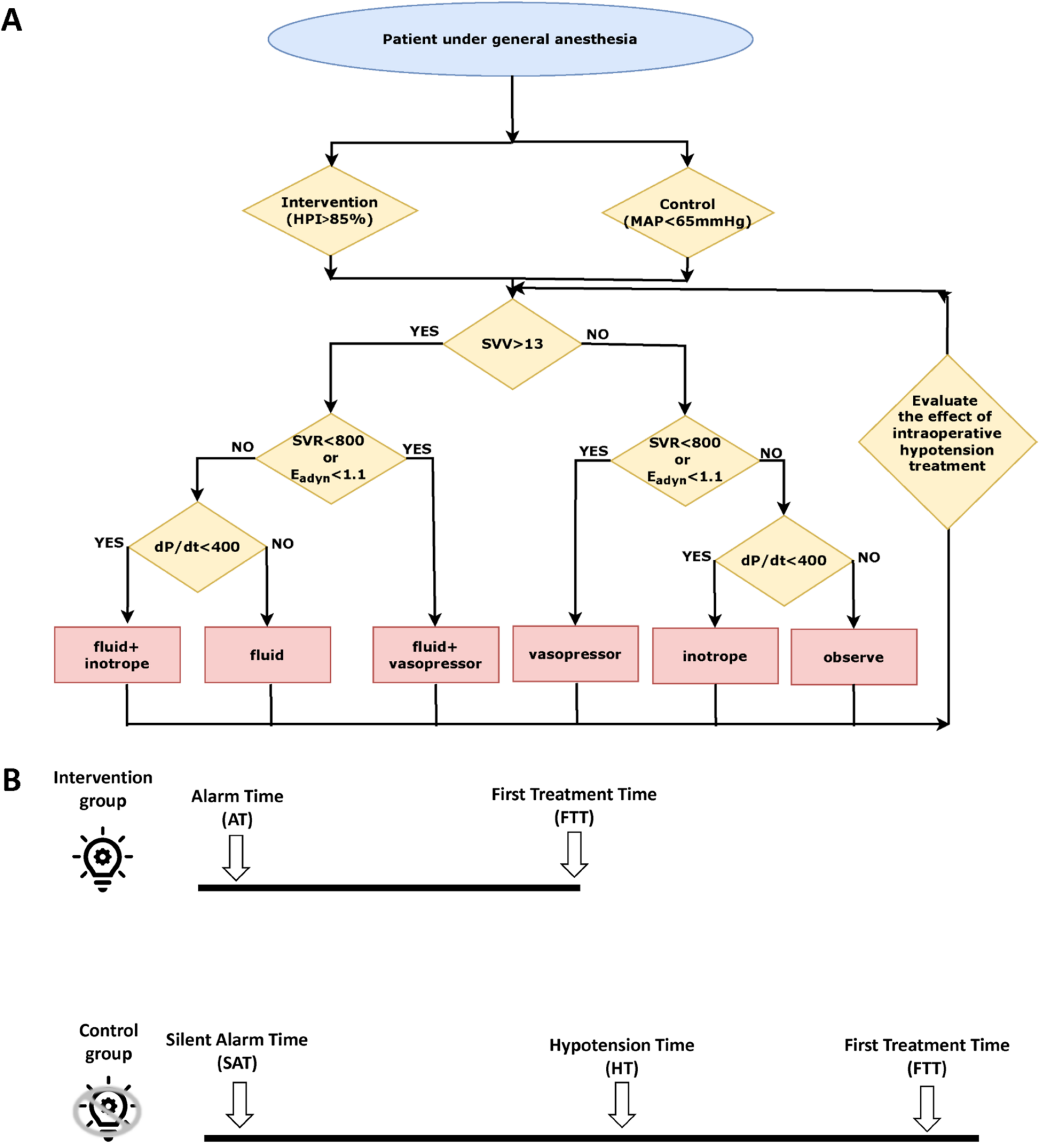
Diagnostic guidance and treatment protocol. **A** Treatment algorithm of intraoperative hypotension management. HPI: Hypotension Prediction Index; MAP: mean arterial pressure; Intervention: HPI guidance; control: no HPI guidance; SVR: systemic vascular resistance; E_adyn_: dynamic arterial elastance; dP/dtmax: systolic slope; **B** Calculation of relevant duration in the intervention and control groups. AT: alarm time; FTT: time of the first intraoperative hypotension treatment; SAT: silent alarm time; HT: hypotension time

Before surgery, the eligible participants were randomized into two groups: one undergoing hemodynamic management with HPI guidance (intervention group) and one being treating according to the standard of care (control group) (Fig. 1A). Computer-generated permutated block randomization (concealed and with varying permutated block sizes of four patients) was applied at a 1:1 allocation ratio, using SAS 9.4 (SAS Institute, Cary, NC, USA). Group allocations were kept in sequentially numbered, opaque, sealed envelopes and were only disclosed to a research assistant not directly involved in the clinical management of the participants or the data collection. To eliminate potential bias, we adopted a single-blinded study design and ensured that the participants were unaware of their group assignment.

### Anesthetic management

The patients underwent standard anesthetic monitoring, which included electrocardiography, pulse oximetry, upper-arm cuff oscillometry, and Bispectral Index (BIS) monitoring, after entering the operating room. General anesthesia was induced with propofol (1.5 -2 mg/kg) and fentanyl (2 μg/kg). Rocuronium was administered at a dosage of 0.7–1.0 mg/kg to assist with tracheal intubation, with additional 10 mg bolus doses administered as needed to sustain muscle relaxation throughout the surgery based on the train of four monitoring. Desflurane was used for maintenance, to achieve a target BIS value between 40 and 60. Mechanical ventilation was administered using a tidal volume of 8 ml per kilogram of predicted body weight, along with a positive end-expiratory pressure of 5 cmH_2_O, and an inspired oxygen fraction adjusted to maintain oxygen saturation at or above 96%. No epidural anesthesia or other regional anesthesia has been used. All patients have been intubated, with each patient equipped with a central venous catheter and an arterial catheter featuring an Acumen IQ sensor (Edwards Lifesciences). All patients were transferred to the intensive care unit (ICU) postoperatively. Extubation was performed base on the same criteria (Popat et al.^2012^) either before leaving the operation room or in the ICU.

### Hypotension prediction index guidance (Intervention) group

All patients were fitted with arterial catheters in the radial artery and connected to the Acumen IQ sensor with the HPI early warning system software (Edwards Lifesciences). Arterial pressure waveform was measured continuously with a sampling frequency of 100 Hz. The HPI early warning system software is installed in a HemoSphere monitor (Edwards Lifesciences), which displays hemodynamic parameters calculated from the arterial waveform every 20 intervals. These values were updated every 20 s. The HPI early warning system generates a visible and audible alarm when the HPI value exceeds 85 (Hatib et al.^2018^), indicating an 85% likelihood that a hypotensive event will occur within the next 15 min (Davies et al.^2020^). The system can also detect the physiological mechanisms that lead to hypotension, including stroke volume variation, dynamic arterial elastance, and systemic vascular resistance (Wijnberge et al.^2020^).

The Acumen IQ sensor pressure transducer was connected to the HemoSphere monitor. The electrical signal generated was also transmitted to the standard monitor in the operating room (Phillips Healthcare, Best, Netherlands), which displayed MAP, systole, diastole, and pulse pressure variation. After placing the arterial catheter, the anesthesiologists visually inspected the arterial waveform signal to detect damping. All anesthesiologists and nurses were informed of the study protocol. A research assistant recorded the medical records related to the surgery and anesthesia procedures. The anesthesiologist and nurse were instructed to take actions within 2 min of an HPI alarm to prevent the occurrence of hypotension. The HemoSphere monitor shows several parameters (including stroke volume variation, dynamic arterial elastance, and systemic vascular resistance) that aided the anesthesiologists in making a differential diagnosis of the underlying cause of the predicted hypotension. The anesthesiologists treated intraoperative hypotension by following one of six treatment options: fluid, vasopressor, inotrope, fluid plus vasopressor, fluid plus inotrope, or observation. The underlying hemodynamic diagnostic guidance and treatment protocol (Fig. 1A) was designed based on a previous published protocol (Maheshwari et al.^2020^).

### Standard-of-care (control) group

In the control group, all patients had arterial catheters inserted in the radial artery and were connected to the Acumen IQ sensor with the HPI early warning system software. The alarm had been activated, but it was muted (silenced), and the relevant visual indicators and screen information were obscured with a cloth covering. All patients were informed that intraoperative hypotension treatment would be initiated at MAP < 65 mmHg and that they would receive the same hemodynamic management to treat the underlying cause of hypotension as the intervention group (Fig. 1A).

### Time-weighted average MAP < 65 mmHg, and other blood pressure related outcomes

The acquisition system of the HPI early warning system software allowed the raw data (HPI and MAP measurements) to be exported to a spreadsheet (Microsoft Excel; Redmond, WA, USA). The data were analyzed in MATLAB 2019b (MathWorks, Natick, MA, USA) (L. Frassanito et al. 2022).

The primary outcome was defined as time weighted average (TWA) MAP < 65 mmHg (Additional File 1, Supplemental Fig. 1). The area under the curve (AUC) of MAP < 65 mmHg was calculated as the depth of hypotension (in mmHg) below a mean arterial pressure of 65 mmHg multiplied by the time in minutes spent below a MAP of 65 mm Hg (mmHg × min). The value of TWA MAP < 65 mmHg was calculated from the value of AUC MAP < 65 mmHg divided by total surgical duration. The formula is as follows:

TWA MAP < 65 mmHg (mmHg) = AUC MAP < 65 mmHg (mm Hg × min) ÷ total surgical duration (min).

We presented an example from one of our patients in Additional File 1, Supplementary Fig. 1–1. The pink area represented the AUC MAP < 65 mmHg, at 852.8 mmHg × min, divided by a total surgical duration of 538 min, yielding a result of 1.59 mmHg. The total duration of MAP < 65 mmHg was 128 min. Other blood pressure related outcomes related to AUC MAP and TWA MAP below the other thresholds (60 mmHg and 55 mmHg) were similarly calculated. The average MAP during surgery and the basal hemodynamic parameters (the first systolic and diastolic blood pressures, MAP, and heart rate in the operation room) were also recorded.

To assess the risk of overtreatment in the intervention group, we examined the occurrence of severe hypertension (MAP > 130 mmHg), reflected as a TWA-MAP above the threshold of 130 mmHg throughout the monitoring period (Schneck et al.^2020^).

### Serious adverse postoperative clinical outcomes

The occurrence of serious adverse postoperative clinical outcomes was recorded based on the Postoperative Morbidity Survey within 30 postoperative days (Maheshwari et al.^2020^). Details of the postoperative complications, based on the Postoperative Morbidity Survey definition, are documented in Supplementary Table 1 of Additional File 1 (Grocott et al.^2007^; Maheshwari et al.^2020^). Death within 30 days of surgery was also recorded.

### Intraoperative outcome recording and clinicians’ actions

We assessed the actions of anesthesiologists in the management of both groups. We recorded (1) treatment options (e.g., vasopressors, inotropic agents, and fluid challenge), (2) cumulative doses of intraoperative medication, (3) the time from the first HPI alarm to the start of hypotension treatment in the intervention group and from the onset of MAP < 65 mmHg to the start of hypotension treatment in the control group (Fig. 1B), and (4) the number of intraoperative hypotension treatments per patient. For (3), in the intervention group, following the previous published method (Maheshwari et al.^2020^), if multiple HPI alarms occurred within 15 min of the initial one, we regarded them as a single event. We would apply the intraoperative hypotension management protocol in our study, using the hemodynamic parameters recorded during the first HPI alarm. If multiple HPI alarms occur within 15 min after the first HPI alarm, we calculated the time difference between the first HPI alarm and the first treatment for hypotension. In the control group, if there were repeated occurrences of MAP < 65 mmHg (hypotension episodes) within 15 min after the initial occurrence, we considered them as one event. We applied the hypotension management protocol, using the hemodynamic parameters recorded during the first hypotension episode. If multiple episodes of hypotension occur within 15 min after the first hypotension episode, we calculated the time difference between the first hypotension episode and the first treatment for hypotension. The time point of the silent alarm was determined post hoc by retrospectively examining the time when MAP < 65 mmHg and calculating the first occurrence of the HPI alarm within fifteen minutes. The time difference between the silence alarm and the treatment of hypotension was obtained. (Fig. 1B) (Wijnberge et al.^2020^).

### Sample size calculation

Following the methodology of a prior study (Wijnberge et al.^2020^), the sample size was determined based on the primary outcome. The primary outcome values for the intervention and control groups were 0.15 and 0.40, respectively, with a standard deviation of 0.32. By setting α and 1-β values at 0.05 and 0.8, the estimated required sample size was calculated as 54 using G Power 3.0. Accounting for a 10% dropout rate, a total of 60 patients were recruited to participate in the study.

### Statistical analysis

For descriptive statistics, we presented continuous data as mean and standard deviation. For highly skewed data, we used median and interquartile range (IQR) instead. We presented categorical data using a contingency table and proportions. For statistical inference, we used the independent T-test and the Mann–Whitney U test to compare the means and medians, respectively. We calculated confidence intervals using the large-sample method for the differences between means and the Hodges-Lehmann method for the differences between medians. We used the chi-squared and Fisher's exact tests to compare the proportions between the groups. A *P* value < 0.05 was considered statistically significant. All analyses followed the intention-to-treat principle and were performed using MATLAB version R2019b (MathWorks) and SPSS version 25 (IBM, Armonk, NY).

## Results

### Study population

**Fig. 2:**
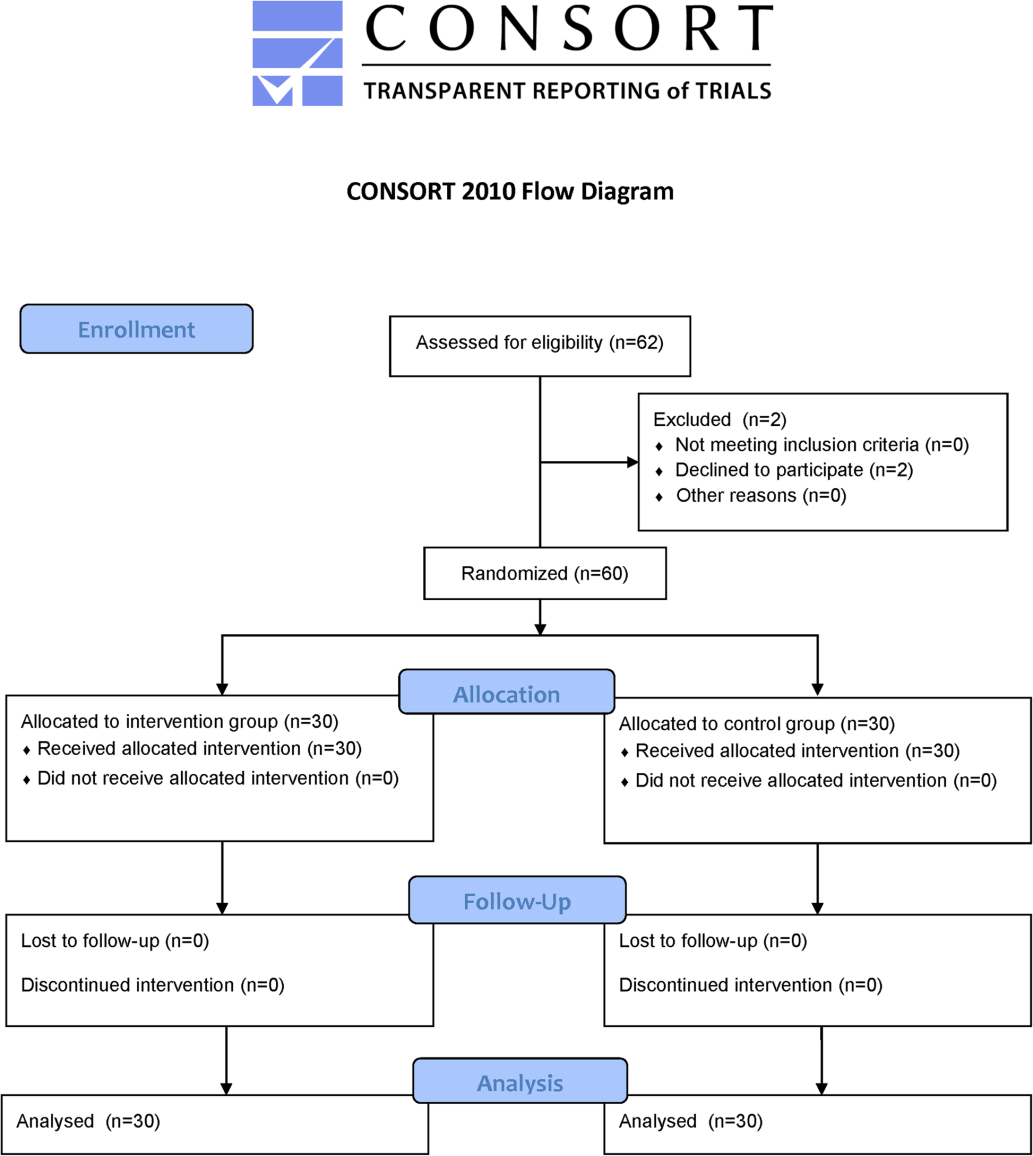
Trial diagram: eligibility, randomization, and participant follow-up

We screened 62 patients, but two declined to participate. We therefore had 60 patients in this study, with 30 patients in the intervention group and 30 patients in the control group. After surgery, we followed all 60 patients for complications and mortality within 30 days. All of them completed the study (Fig. 2), and all had arterial catheters successfully inserted for monitoring, with no intraoperative monitor malfunctions. No intraoperative issues with arterial catheter monitoring or equipment, such as equipment malfunction, have been noted. The median patient age was 62 years [Q1, Q3: 53 to 68]; 48 (80%) of them were men. The median surgical duration was 489.50 min [Q1, Q3: 320.75, 638]. Table 1 displays the baseline characteristics of the patients enrolled in the study. The basal hemodynamic parameters were comparable between the two groups (Additional file 1, Supplementary Table 2).

**Table 1:** Baseline characteristics

	**Intervention (***n* **= 30)**	**Control (***n* **= 30)**	***P* value**
Age, years	60.17 ± 10.6	59.70 ± 10.3	0.86
Men, *n* (%)	23 (76.7%)	23 (76.7%)	0.51
Smoking, *n* (%)	6 (35.3%)	11 (36.7%)	0.15
Drinking, *n* (%)	5 (16.7%)	8 (26.7%)	0.34
Chewing betel nuts, *n* (%)	2 (6.7%)	5 (16.7%)	0.42^§^
Hypertension, *n* (%)	18 (60.0%)	12 (40.0%)	0.12
Diabetes, *n* (%)	3 (10.0%)	6 (20.0%)	0.47^§^
CAD, *n* (%)	1 (3.3%)	1 (3.3%)	1.00^§^
COPD, *n* (%)	0 (0.0%)	0 (0.0%)	
CVA, *n* (%)	1 (3.3%)	0 (0.0%)	1.00^§^
CKD, *n* (%)	0 (0.0%)	0 (0.0%)	
Body mass index, kg·m^−2^	22.2 ± 5.1	21.7 ± 4.5	0.67
Creatinine	0.80 [0.6, 1.0]	0.90 [0.7, 1.1]	0.22
eGFR	103.4 [80.3, 122.5]	92.8 [72.6, 102.7]	0.11
ASA status			0.49
II, *n* (%)	11 (36.7%)	15 (50.0%)	
III, *n* (%)	19 (63.3%)	15 (50.0%)	
Surgery			0.43
Pancreas, *n* (%)	12 (40.0%)	8 (26.7%)	
Oral cancer, *n* (%)	11 (36.7%)	11 (36.7%)	
Esophagus, *n* (%)	7 (23.3%)	11 (36.7%)	
Surgical duration, min	517.5 ± 222.1	491.4 ± 206.1	0.64

^§^The Fisher exact test was used of the Chi-squire test due to 50% of the cells have expected counts less than 5

Data are presented as mean ± standard deviation, median [Q1, Q3], or percentage (%); intervention: Hypotension Prediction Index *HPI* guidance, Control standard-of-care treatment, no *HPI* guidance, *CAD* coronary arterial disease, *COPD* chronic obstructive pulmonary disease, *CVA* cardiovascular accident, *CKD* chronic kidney disease, *eGFR* estimated Glomerular filtration rate, *ASA* The American Society of Anesthesiologists

## Intraoperative parameters associated with TWA MAP

The median TWA MAP < 65 mmHg, which was the primary outcome, was 0.017 mmHg [Q1, Q3: 0.001, 0.077 mmHg] in the intervention group and 0.37 mmHg [Q1, Q3: 0.20, 0.58 mmHg] in the control group, with a median difference of 0.32 mmHg (95% CI, 0.23, 0.42; *P* < 0.001) (Table 2). The AUC was lower and the time spent at a MAP < 65 mmHg was significantly shorter in the intervention group than the control group (Table 2). The distributions of the TWA MAP and the duration of MAP < 65 mmHg were shown in Additional File 1, Supplementary Figs. 1–2 and 1–3, respectively. These differences between the groups were also significant when we used MAP thresholds of < 60 mmHg or < 55 mmHg (Table 2). Further, the median MAP was 87.54 mmHg [Q1, Q3: 85.08, 93.46 mmHg] in the intervention group, and 77.92 mmHg [Q1, Q3: 74.56, 83.82 mmHg] in the control group (*P* < 0.001). There was no difference between the two groups in terms of the severe hypertension (Additional File 1, Supplementary Table 4).

**Table 2:** Outcomes associated with TWA-MAP and clinician behaviors

	**Intervention (***n* **= 30)**	**Control (***n* **= 30)**	**Median difference (95% CI)**	***P* value**
**TWA-MAP associated outcomes**				
**Primary outcome**				
TWA-MAP < 65 mmHg, mmHg	0.02 [0.003,0.08]	0.37 [0.20,0.58]	0.32 (0.23, 0.42)	< 0.001
**Secondary outcomes**				
AUC < 65 mmHg, mmHg$\times$min	11.91 [2.43,42.82]	230.95 [129.20,375.87]	200.80 (135.80, 282.90)	< 0.001
Duration of MAP < 65 mmHg, min	3.05 [1.60, 9.44]	59.47 [28.91, 120.45]	54.12 (35.89, 68.75)	< 0.001
TWA-MAP < 60 mmHg, mmHg	0.001 [0.00, 0.02]	0.08 [0.04, 0.18]	0.08 (0.04, 0.12)	< 0.001
AUC < 60 mmHg, mmHg$\times$min	0.75 [0.00, 8.57]	43.67 [20.87, 106.52]	40.18 (22.30, 80.32)	< 0.001
Duration of MAP < 60 mmHg, min	0.75 [0.00, 8.57]	13.16 [8.80, 26.99]	12.52 (9.32, 16.40)	< 0.001
TWA-MAP < 55 mmHg, mmHg	0.00 [0.00,0.003]	0.01 [0.00,0.03]	0.01 (0.001, 0.02)	< 0.001
AUC < 55 mmHg, mmHg$\times$min	0.00 [0.00,0.62]	5.14 [0.14,25.45]	4.91 (0.67, 15.47)	< 0.001
Duration of MAP < 55 mmHg, min	0.00 [0.00, 0.50]	3.28 [0.30, 8.79]	2.85 (0.66, 5.62)	< 0.001
**Clinician behaviours**				
Number of treatments per patient	7.5 [4.0, 12.5]	9.5 [4.0, 17.0]	1.00 (-2.00, 5.00)	0.48
Duration from AT or HT to FTT, s	42.8 [20.4, 79.0]	41.2 [35.5, 47.5]	-4.27 (-19.04, 10.90)	0.59
Duration from AT or SAT to FTT, s	42.8 [20.4, 79.0]	200.2 [99.7, 261.8]	144.76(93.87, 196.35)	< 0.001

Data are presented as median [Q1, Q3]. *Intervention* hypotension prediction index, *HPI* guidance, control no *HPI* guidance, *TWA-MAP* time-weighted average—mean arterial pressure, *AUC* the area under the curve of mean arterial pressure, *MAP* mean arterial pressure, *AT* alarm time, *HT* hypotension time, *SAT* silent alarm time, *FTT* first intraoperative hypotension treatment time, *CI* confidence interval, The median difference and 95% CI were estimated using Hodges–Lehmann estimator

### Intraoperative outcomes and serious adverse postoperative clinical outcomes within 30 postoperative days

The estimated blood loss, urine output, and infusion volume including crystalloid, colloid, blood transfusion including RBC, platelet, and fresh frozen plasma (FFP) have been shown in the Table 3. Most of the above factors showed significant differences between the intervention and control groups, except for crystalloid and FFP. The fluid balance at the end of surgery, indicating the difference between input and output fluids, didn't differ significantly between the two groups. Additionally, the intraoperative medication presented no significant difference (Table 3).

**Table 3:** Intraoperative outcomes

Outcome	Intervention (*n* = 30)	Control (*n* = 30)	Difference (95% CI)	*P* value
Crystalloids, mL	2913.6 ± 1767.5	2718.9 ± 1034.2	-194.7 (-943.1, 553.7)^†^	0.61
Colloid, mL	500 [500, 1100]	100 [0, 500]	-500 (-900, -400)^∥^	< 0.001
Urine output, mL	1600 [937.5, 2287.5]	700 [375, 1012.5]	-895 (-1260, -550)^∥^	< 0.001
Estimated blood loss, mL	500 [287.5, 640]	200 [137.5, 412.5]	-200 (-300, -100)^∥^	0.002
RBC transfusion, U	4 [1, 5]	0.5 [0, 2]	-2 (-4, -1) ^∥^	< 0.001
Platelet transfusion, U	0 [0, 3]	0 [0, 0]	0 (0, 0) ^∥^	0.005
FFP transfusion, U	0 [0, 6]	0 [0, 0.5]	0 (0, 0) ^∥^	0.15
Fluid balance at the end of surgery, mL	1404.6 ± 1512.5	1810.7 ± 983.9	406.1 (-253.3, 1065.6)^†^	0.22
Cardiac index	3.6 ± 0.7	3.2 ± 0.7	-0.4 (-0.7, 0.00)^†^	0.051
Stroke volume	73.9 ± 19.1	68.4 ± 12.8	- 5.5 (-13.9, 3.0)^†^	0.20
Norepinephrine, μg	71.5 [15.0, 384.3]	45.00 [0.0, 141.0]	-18.00 (-80.0, 15.0)^∥^	0.20
Fentanyl, μg	242.5 [200.0, 400.0]	212.5 [163.8, 418.8]	0.0 (-100.0, 50.0)^∥^	0.63
Ephedrine, *n* (%)	5 (16.7%)	6 (20.0%)	-3.3% (-26.2, 19.6)^††^	1.00
Nicardipine, *n* (%)	9 (30.0%)	6 (20.0%)	10.0% (-15.1, 35.1)^††^	0.55
Labetalol, *n* (%)	4 (13.0%)	3 (10.0%)	3.3% (-16.2, 22.9)^††^	1.00
Morphine, *n* (%)	8 (26.7%)	8 (26.7%)	0.0% (-22.4, 22.4)^††^	1.00

Data are presented as mean ± standard deviation or median [Q1, Q3] or percentage (%). *Intervention* Hypotension Prediction Index *HPI* guidance, control: standard-of-care treatment, no *HPI* guidance; fluid balance at the end of surgery: input fluid minus output fluid, *FFP* Fresh Frozen Plasma, The median difference and 95% confidence interval *CI* were estimated using Hodges–Lehmann estimator; †: mean difference; ∥:median difference; ††: proportional difference

The median length of intensive care unit stay was 4.0 days in the intervention group and 4.5 days in the control group (*P* = 0.97, Table 4). The median length of hospitalization was 20.5 days in the intervention group and 21.5 days in the control group (*P* = 0.75, Table 4). The Table 4 shows the complications according to the Postoperative Morbidity Survey definition. No statistically significant differences in postoperative complications were found between the two groups. The incidence of gastrointestinal complications was numerically different between groups, however, this difference was not statistically significant after applying the Bonferroni correction for performing multiple comparisons. There were no adverse events leading to death in the intervention group while there was one (3.3%) in the control group (postoperative pneumonia).

**Table 4:** Postoperative outcomes

Outcome	Intervention (*n* = 30)	Control (*n* = 30)	*P* value
**Days in hospital and ICU**			
ICU stay, days	4.0 [2.8, 5.2]	4.5 [2.8, 5.0]	0.97
Hospitalization, days	20.5 [16.0, 27.5]	21.5 [17.8, 27.8]	0.75
**Postoperative complications based on POMS**			
Pulmonary complications, *n* (%)	19 (63.3%)	20 (66.7%)	0.79
Cardiovascular complications, *n* (%)	0 (0.0%)	3 (10.0%)	0.24
Gastrointestinal symptoms, *n* (%)	6 (20.0%)	14 (46.7%)	0.028
Infection, *n* (%)	3 (6.67%)	6 (20.0%)	0.47
Renal complications, *n* (%)	0(0.0%)	2 (6.67%)	0.49
Neurological complications, *n* (%)	0 (0.0%)	0 (0.0%)	
Wound complications, *n* (%)	0 (10.0%)	0 (0.0%)	
Blood transfusion requirement, *n* (%)	1 (3.33%)	1 (3.33%)	1.0
New onset pain, *n* (%)	0 (0.00%)	1 (3.33%)	1.0
Adverse event causing to death, *n* (%)	0 (0.0%)	1 (3.33%)	1.0

Data are presented as median [Q1, Q3] and percentage (%). Intervention Hypotension Prediction Index, *HPI* guidance control: standard-of-care treatment, no *HPI* guidance, *ICU* intensive care unit, *POMS* Postoperative Morbidity Survey (see detailed definitions in Supplementary Table 1)

### Clinician actions

The median number of treatments per patient was comparable between groups ( *P* = 0.48, Table 2). The median duration from the time of the alarm (intervention group) or at which hypotension was recorded (control group) to the first treatment also did not differ significantly between groups (*P* = 0.59, Table 2). However, the post hoc analysis indicated that the median duration from alarm to first treatment was significantly shorter in the intervention group (42.78 s) than that from the silent alarm to first treatment in the control group (200.16 s). There were no statistically significant differences in treatment choice for intraoperative hypotension between the groups (Additional file 1, Supplementary Table 3). The most common choice of intraoperative hypotension treatment in both groups was vasopressors.

## Discussion

Our study demonstrated that using HPI guidance for hemodynamic management in patients undergoing general anesthesia can effectively decrease the severity of hypotension. This was measured by TWA MAP < 65, 60, and 55 mmHg, as well as the median MAP during the surgery.

Hemodynamic management with HPI guidance led to a statistically significant reduction in the severity of hypotension in our patient sample, which is in line with previous research (Tsoumpa et al.^2021^; Wijnberge et al.^2020^). However, our study's results contrast with those of a randomized trial that found no statistically significant difference in the duration or severity of intraoperative hypotension between patients treated with and without HPI guidance (Maheshwari et al.^2020^). There are two potential explanations for this discrepancy between our study and that by Maheshwari et al. Firstly, in the Maheshwari et al. study, the clinicians in the control group, where the HPI monitor screen was completely covered with a cloth and the alarm sounds was silenced, may have implemented more proactive approaches as a compensatory measure to mitigate the risk of hypotension. In contrast, in our study's control group, we only partially covered the HPI monitor screen. The clinicians were still able to view some of the hemodynamic parameters, so they may not have been as proactive in managing potential hypotension. They initiated treatment for hypotension only when MAP was below 65 mmHg. Secondly, our study included younger patients than the Maheshwari et al. study, despite both studies focusing on non-cardiac surgery. This difference in patient demographics, particularly in terms of American Society of Anesthesiologists status severity, may have contributed to the inconsistent findings.

Our postoperative complication results were consistent with previous studies (Luciano Frassanito et al.^2023^; Maheshwari et al.^2020^; Murabito et al.^2022^; Tsoumpa et al.^2021^; Wijnberge et al.^2020^; Yoshikawa et al.^2024^), which showed no significant differences between intervention and control groups. Additionally, we have concerns about the distribution of drinking habits, diabetes, and types of surgery. In our study, the intervention group had lower rates of drinking, prevalence of diabetes, and esophageal surgery than the control group. These differences were not statistically significant, but this may have been due to our small sample size. Further research with a larger sample size is thus necessary to validate our findings. We found no significant differences between the two groups in terms of the duration from the alarm (intervention group) or the occurrence of hypotension (control group) to the first treatment. This indicates that the anesthesiologists’ response time to the HPI guidance was similar to the time needed to manually detect intraoperative hypotension and initiate treatment. However, we observed a shorter duration from the alarm to the first treatment in the intervention group than from the silenced alarm to the first treatment in the control group. This suggested that using HPI guidance provides a greater opportunity for timely intervention and effective management of intraoperative hypotension.

We believe that our study is indicative of the advancements made in using HPI guidance to initiate early management of intraoperative hypotension. HPI -guided hemodynamic management and care approaches offer significant convenience, allowing clinicians to respond promptly to intraoperative hypotension management based on HPI alarms during the monitoring process. Based on our findings, early initiation of intraoperative hypotension treatment following HPI guidance appears to be effective in preventing intraoperative hypotension. To determine the impact of HPI technology on postoperative outcomes in major surgeries, further studies are required in the future.

### Limitations

Our study had several limitations that should be considered. Firstly, our trial was limited to a 30-day observation period. To gain a better understanding of the long-term incidences of complications after surgery, future studies could consider extending the tracking duration to six months or even one year. Secondly, the inclusion of patients exclusively from a tertiary medical center and the exclusion of patients with certain cardiac conditions or major organ dysfunction may limit the generalizability of our findings.

## Conclusions

In this randomized controlled trial, following HPI guidance led to a decrease in intraoperative hypotension. Further investigation is required to evaluate the effect of HPI guidance on postoperative complications and mortality.

### Supplementary Information

Supplementary Material 1.

## Data Availability

Data are available from the authors upon request.
