# Supplementary material for: Hypotension prediction index for prevention of intraoperative hypotension in patients undergoing general anesthesia: a randomized controlled trial
Source: Perioper Med (Lond). 2024 Jun 15;13:57. doi: 10.1186/s13741-024-00414-7 (PMC11180403; doi:10.1186/s13741-024-00414-7)
Supplement: Supplementary file 1 — Supplementary Material 1. [file 13741_2024_414_MOESM1_ESM.docx]

**ADDITIONAL FILES**

[Supplementary Table 1. Definitions of the Post-operative Morbidity Survey 2](#_Toc167963889)

[Supplementary Table 2 Basal hemodynamic parameters between the two groups 3](#_Toc167963890)

[Supplementary Table 3. Treatment choice comparison between the intervention and control groups 4](#_Toc167963891)

[Supplementary Table 4. Severe hypertension of TWA MAP associated outcomes 5](#_Toc167963892)

[Supplementary Figure 1-1: Time-weighted average mean arterial pressure < 65mmHg, calculated for one patient. 6](#_Toc167963893)

[Supplementary Figure 1-2. Time-weighted average mean arterial pressure < 65mmHg between the intervention and control groups 7](#_Toc167963894)

[Supplementary Figure 1-3. The duration of mean arterial pressure <65 mmHg between the intervention and control groups 8](#_Toc167963895)

# Supplementary Table 1. Definitions of the Post-operative Morbidity Survey

| **Complications** | **Criteria** | **Source of data** |
| --- | --- | --- |
| **Pulmonary complications** | New requirement of oxygen or respiratory support | Patient observation and treatment chart |
| **Infection** | Temperature >38 °C in the last 24 h and newly prescribed antibiotics | Treatment chart and observation chart |
| **Kidney injury** | Oliguria (500 mL/24 h) noted or increased serum creatinine (>30 % from preoperative level) | Patient observation, fluid balance chart, and biochemistry result |
| **Gastrointestinal complications** | Patient cannot tolerate oral or enteral diet; nausea, vomiting, and abdominal distension | Patient questioning, fluid balance chart, and treatment chart |
| **Cardiovascular complications** | Diagnostic tests/therapy within the last 24 h, showing new myocardial infarction or ischemia, atrial or ventricular arrhythmias, cardiogenic pulmonary edema or thrombotic events | Treatment chart and note review |
| **Neurological complications** | New focal deficit delirium or coma | Note review and patient questioning |
| **Wound complications** | Operation wound requires surgical exploration or drainage of pus | Note review and pathology result |
| **Hematological requirement** | Requirement for blood transfusion within the last 24 h | Treatment chart and fluid balance chart |
| **New-onset pain** | New-onset severe postoperative pain that requires a new strong opioid or new nerve block. | Treatment chart and patient questioning |

The Post-operative Morbidity Survey was based on Grocott et al., *J Clin Epidemiol.*, 2007.

# Supplementary Table 2 Basal hemodynamic parameters between the two groups

|  | **Intervention (n=30)** | **Control (n=30)** | **P value** |
| --- | --- | --- | --- |
| **SBP, mmHg** | 123 [114, 137] | 122 [109, 150] | 0.94 |
| **DBP, mmHg** | 77 [70, 84] | 78 [70, 84] | 0.83 |
| **MAP, mmHg** | 92 [87, 101] | 93 [82, 106] | 0.86 |
| **HR, beats/min** | 83 [74, 91] | 78 [69, 88] | 0.27 |

SBP: Systolic blood pressure; DBP: diastolic blood pressure; MAP: mean arterial pressure; HR: heart rate; intervention: hypotension prediction index (HPI) guidance; control: standard of care, no HPI guidance

# Supplementary Table 3. Treatment choice comparison between the intervention and control groups

| **Treatment, total times** | **Intervention (n = 279)** | **Control (n = 307)** | ***P* value** |
| --- | --- | --- | --- |
| Fluid + vasopressor | 59 | 58 | 1.00 |
| Fluid + inotropic agent | 9 | 3 | 0.60 |
| Fluid | 22 | 32 | 0.23 |
| Vasopressor | 172 | 195 | 0.44 |
| Inotropic agent | 5 | 9 | 0.65 |
| Observation | 12 | 10 | 0.95 |

Intervention: Hypotension Prediction Index (HPI) guidance; control: standard of care, no HPI guidance; n: total treatment times. P value calculated using the Mann-Whitney U test.

# Supplementary Table 4. Severe hypertension of TWA MAP associated outcomes

|  | **Intervention (n=30)** | **Control (n=30)** | **Median difference (95% CI)** | ***P* value** |
| --- | --- | --- | --- | --- |
| TWA-MAP > 130 mmHg, mmHg | 0.008 [0, 0.025] | 0 [0, 0.012] | 0 [-0.01, 0] | 0.09 |
| AUC > 130 mmHg, mmHg$\times$min | 3.74 [0, 15.76] | 0 [0, 5.15] | -0.85 [-5.56, 0] | 0.09 |
| Duration of MAP > 130 mmHg, min | 0.93 [0, 2.49] | 0 [0, 1.27] | -0.09 [-1.08, 0] | 0.09 |

Data are presented as median [Q1, Q3]. Intervention: hypotension prediction index (HPI) guidance; control: standard of care, no HPI guidance; TWA-MAP: time-weighted average - mean arterial pressure; AUC: the area under the curve of mean arterial pressure; MAP, mean arterial pressure; CI: confidence interval; The median difference and 95% CI were estimated using Hodges–Lehmann estimator.

# Supplementary Figure 1-1: Time-weighted average mean arterial pressure < 65mmHg, calculated for one patient.

**
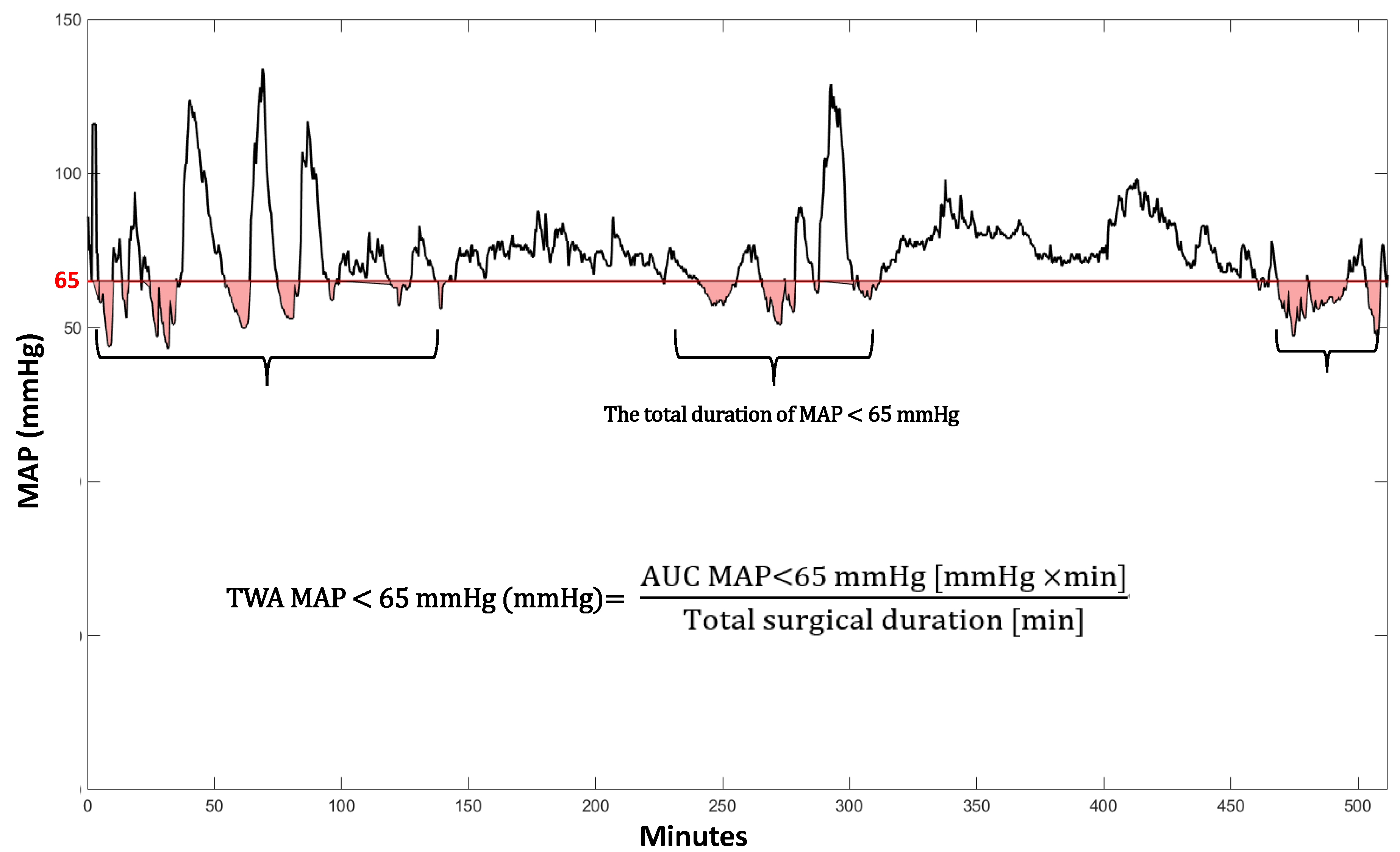
**

Abbreviation: MAP: mean arterial pressure; TWA MAP: time-weighted average mean arterial pressure; AUC MAP: The area under the curve of mean arterial pressure

The pink area = the area under the curve (AUC) of mean arterial pressure (MAP) < 65mmHg.

The total AUC of MAP< 65mmHg = 852.8 mmHg × min.

The total surgical length = 538 min

The time-weighted average MAP < 65mmHg = $\frac{The total AUC of MAP<65mmHg [mmHg \times min]}{The total surgical length [min]}$= $\frac{852.8 [mmHg\times min]}{538 [min]}$ = 1.59 mmHg

The total duration of MAP < 65 mmHg = 128 min

# Supplementary Figure 1-2. Time-weighted average mean arterial pressure < 65mmHg between the intervention and control groups

**
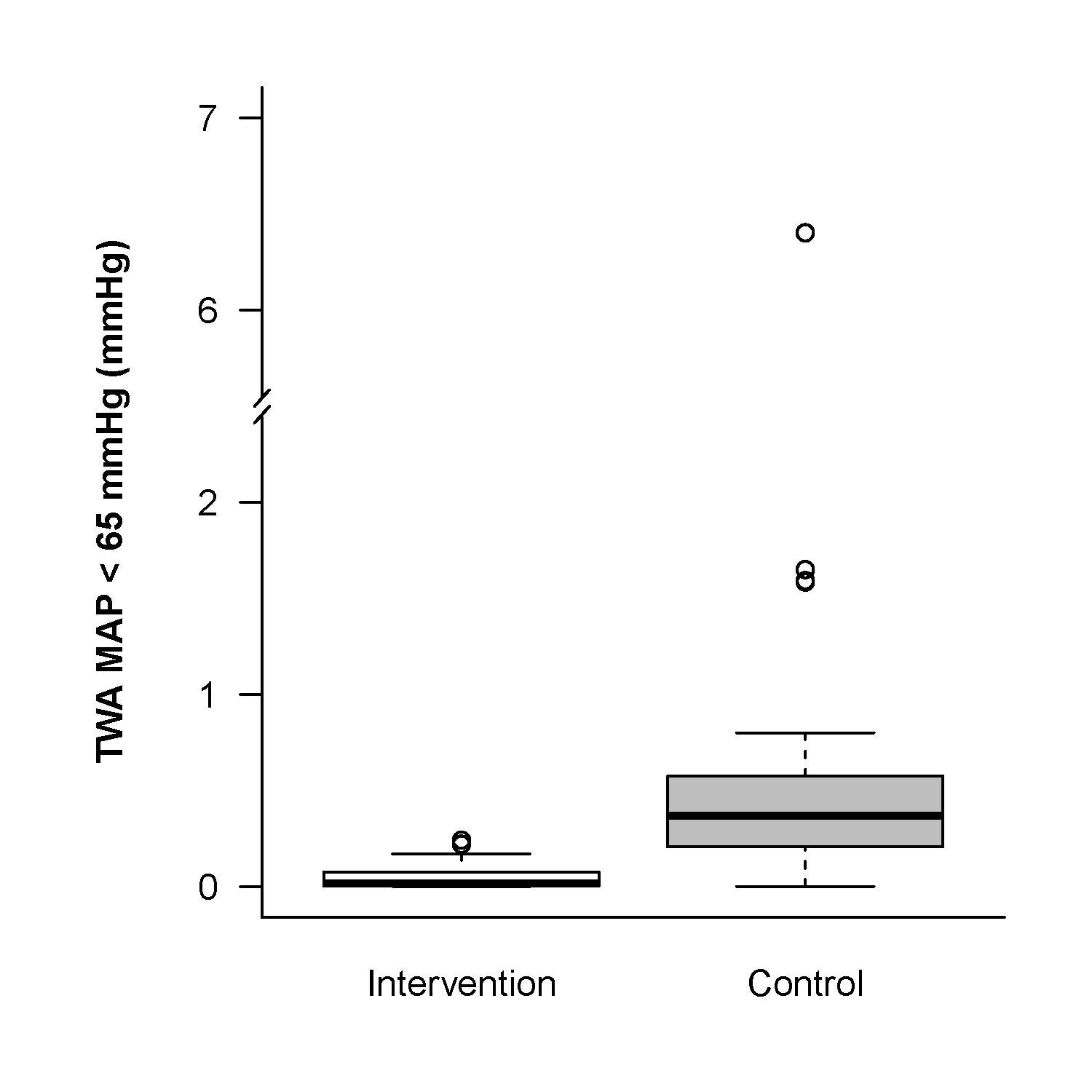
**

Intervention: Hypotension Prediction Index (HPI) guidance; control: standard-of-care treatment, no HPI guidance; TWA MAP: time-weighted average mean arterial pressure

# Supplementary Figure 1-3. The duration of mean arterial pressure <65 mmHg between the intervention and control groups


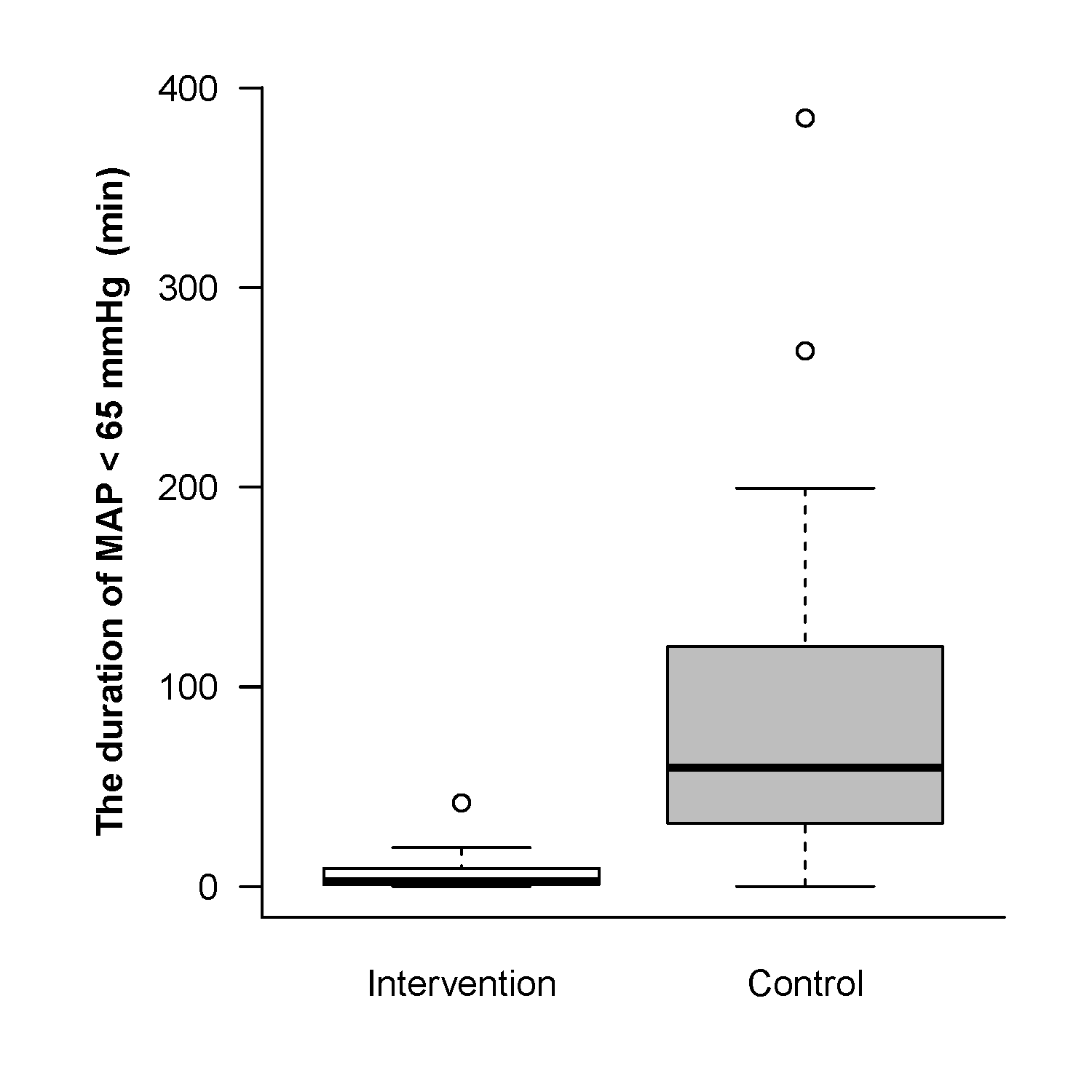


Intervention: Hypotension Prediction Index (HPI) guidance; control: standard-of-care treatment, no HPI guidance; MAP: mean arterial pressure
